# Transcriptomic response of *Campylobacter jejuni* following exposure to acidified sodium chlorite

**DOI:** 10.1038/s41538-021-00103-5

**Published:** 2021-08-02

**Authors:** Gayani Weerasooriya, Andrea R. McWhorter, Samiullah Khan, Kapil K. Chousalkar

**Affiliations:** School of Animal and Veterinary Sciences, University of Adelaide, Roseworthy, SA Australia

**Keywords:** Applied microbiology, Antimicrobial resistance

## Abstract

Chemical decontamination during processing is used in many countries to mitigate the *Campylobacter* load on chicken meat. Chlorine is a commonly used sanitizer in poultry processing to limit foodborne bacterial pathogens but its efficacy is limited by high bacterial loads and organic material. Acidified sodium chlorite (ASC) is a potential alternative for poultry meat sanitization but little is known about its effects on the cellular response of *Campylobacter*. In this study, the sensitivity of *C. jejuni* isolates to ASC was established. RNAseq was performed to characterize the transcriptomic response of *C. jejuni* following exposure to either chlorine or ASC. Following chlorine exposure, *C. jejuni* induced an adaptive stress response mechanism. In contrast, exposure to ASC induced higher oxidative damage and cellular death by inhibiting all vital metabolic pathways and upregulating the genes involved in DNA damage and repair. The transcriptional changes in *C. jejuni* in response to ASC exposure suggest its potential as an effective sanitizer for use in the chicken meat industry.

## Introduction

*Campylobacter* spp. are the leading bacterial cause of foodborne gastroenteritis in humans^1,2^. Raw and improperly handled chicken meat are two of the most commonly identified sources of *Campylobacter* during traceback investigations; therefore, reducing *Campylobacter* levels on raw poultry meat is an important aspect of food safety^3^. *Campylobacter* spp. often colonize the intestinal tract of broiler chickens and during processing, carcasses and meat pieces can become contaminated^4^. Globally, the prevalence of *Campylobacter* contaminated poultry carcasses ranges between 60 and 80%^5^. Improper food handling procedures and poor kitchen hygiene measures can increase the risk of cross-contamination^6^.

The manufacturing of poultry products is highly regulated and many regulatory agencies recommend the use of generally recognized as safe (GRAS) sanitizers to mitigate *Campylobacter* in the food supply chain^7,8^. GRAS chemicals, such as chlorine, chlorine dioxide (ClO_2_), sodium hypochlorite (SH), acidified sodium chlorite (ASC), peracetic acid (PAA), and ozone have been recommended for use in processing plants in countries including Australia, New Zealand^9^, and the USA^10^. The European Union does not permit the use of chlorine during processing due to health and safety issues^11^.

Historically, chlorine has been the most commonly used sanitizer in poultry meat processing in the USA, Australia, New Zealand, and most Asian countries^12–14^. Despite its widespread use, it is less effective than ASC, PAA, or trisodium phosphate in reducing *Campylobacter* levels on chicken meat^12,15^. At concentrations widely used in the poultry meat industry, chlorine induces sublethal injury that is significantly impacted by bacterial load and organic matter content^16^. Considering the limitations of chlorine, ASC has been tested by several groups as an alternative to reduce *Campylobacter* contamination on carcasses^14,17^.

Several countries in Europe, Canada, and Australia recommend the use of ASC (<1200 ppm sodium chlorite, pH 2.3–2.9) for chemical decontamination in the food industry^18,19^. ASC is an oxidative broad-spectrum antimicrobial, which generates chlorous acid that gradually decomposes into chloride ion, chlorate ion, chlorine, and ClO_2_^20^. It has been hypothesized that uncharged chlorous acid penetrates the bacterial cell membrane and disrupts protein synthesis by reacting with sulfhydryl, sulfide, and disulfide in nucleotides^21^.

Factors that potentially affect the bactericidal activity of ASC, however, have not been explored. The chemical decontamination of chicken carcasses, for example, generally occurs at low temperatures (<5 °C) to prevent bacterial replication and in the presence of organic matter. In the present study, we investigated the in vitro susceptibility of *Campylobacter jejuni (C. jejuni)* over a range of ASC concentrations. Transcriptional analysis of *C. jejuni* following exposure to chlorine and ASC was conducted to characterize the bacterial response to these chemicals.

## Results

### Bactericidal activity of acidified sodium chlorite on *Campylobacter jejuni*

**Fig. 1:**
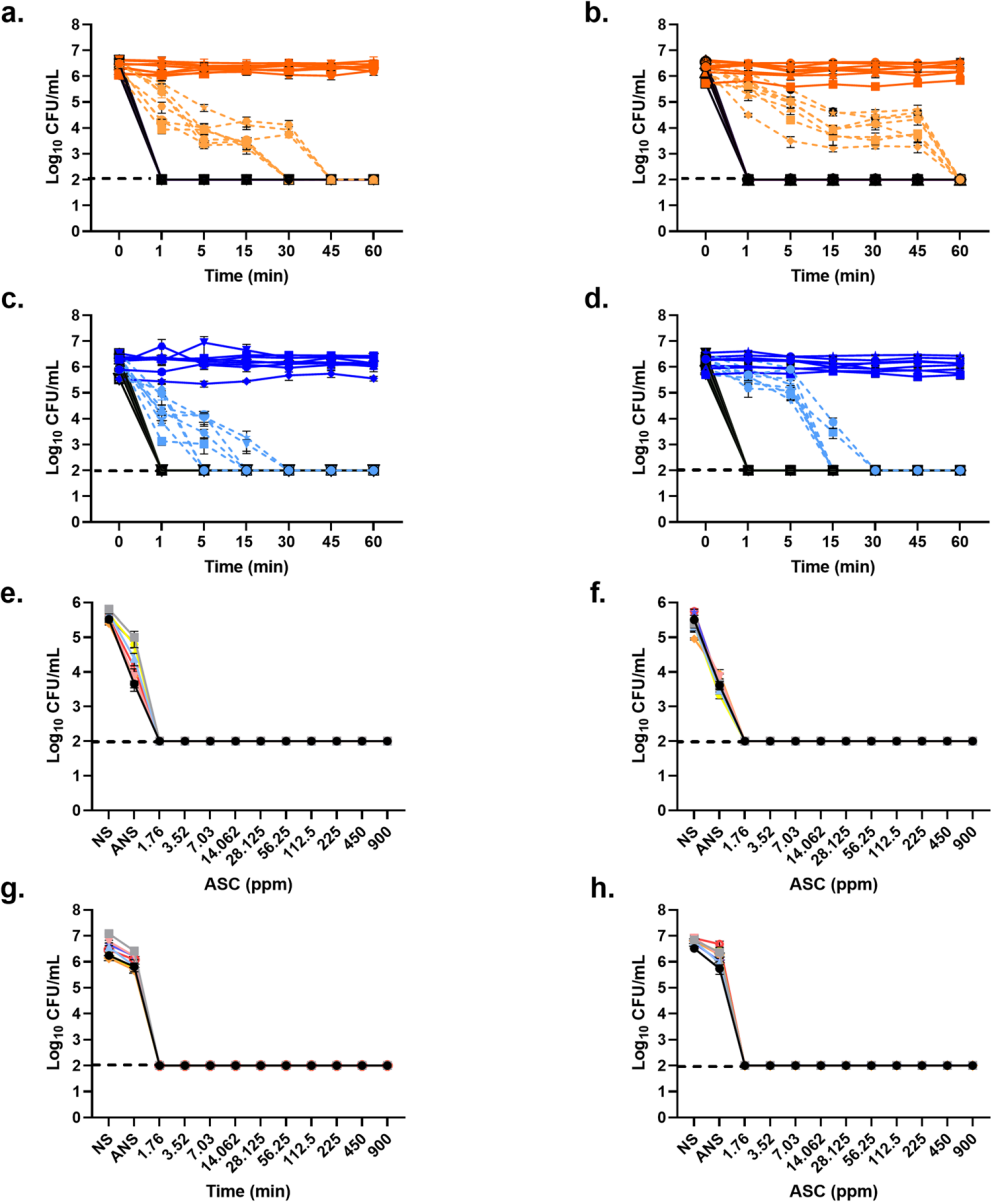
Bactericidal kinetics of *C. jejuni* with ASC. In time-kill assays (**a**, **b**, **c**, **d**), ATCC *C. jejuni* 33221 and seven chicken meat isolated *C. jejuni* were exposed to ASC (900, 450, 225 ppm) for 60 min. Broth control (dark orange lines), acidified broth control (light orange hash lines), 0.9% saline control (dark blue lines), and acidified 0.9% saline control (light blue hash lines) were compared with ASC treated groups (black lines). Temperature effect was compared at 5 °C (**a**, **c**) or 25 °C (**b**, **d**). In inactivation assays (**e**, **f**, **g**, **h**), the same *C. jejuni* isolates with ATCC strain were exposed to ASC (1.76–900 ppm) for 1 min. Inactivation kinetics were determined for both 10^6^ CFU/mL (**e**, **f**) at 5 °C (**e**) and 25 °C (**f**) and the 10^8^ CFU/mL (**g**, **h**) at 5 °C (**g**) and 25 °C (**h**). Data are presented as mean log_10_ CFU/mL ± SEM. The dotted line indicates the limit of detection of culturable bacteria. (NS; 0.9% saline, ANS; acidified 0.9% saline).

All *C. jejuni* isolates (*n* = 89) exhibited higher sensitivity to ASC. For all isolates, both the minimum inhibitory concentration (MIC) and minimum bactericidal concentration (MBC) values were 7.03 ppm. Time-kill curves confirmed the sensitivity of the *C. jejuni* isolates. Following exposure to ASC, none of the isolates were cultured from any of the ASC concentrations tested (900–1.76 ppm) (Fig. 1a, b, c, d). No significant variation in bacterial titre was observed over time for *C. jejuni* isolates suspended in either nutrient broth no. 2 (NB2) (Fig. 1a, b) or 0.9% saline (Fig. 1c, d). To understand the role of pH in inactivation, *C. jejuni* isolates were also exposed to acidified 0.9% saline or NB2 (pH 2.5). Culturability of the *C. jejuni* isolates significantly (*P* < 0.0001) declined over time both at 5 and 25 °C. All the *C. jejuni* isolates declined to the minimum detection level at 25 °C in the acidified NB2 within 45 min, while all isolates were inactivated within 60 min at 5 °C. *C. jejuni* exhibited higher susceptibility to acidified 0.9% saline, while the culturability was completely inhibited after 30 min at both temperatures.

ASC exhibited a strong antimicrobial effect against all *C. jejuni* isolates at both 5 and 25 °C (Fig. 1e, f, g, h). After a 1-min exposure, no culturable bacteria were observed in any of the ASC concentrations tested. Bacterial load did not affect the efficacy of ASC. Aliquots of non-culturable *C. jejuni* were transferred to Preston broth (resuscitation media) to characterize the recovery potential following ASC exposure. None of the inactivated *C. jejuni* isolates at either bacterial concentration (10^6^ or 10^8^ colony-forming unit (CFU)/mL) were resuscitated.

### Transcriptome profiling of *C. jejuni* post-exposure to chlorine and ASC

**Fig. 2:**
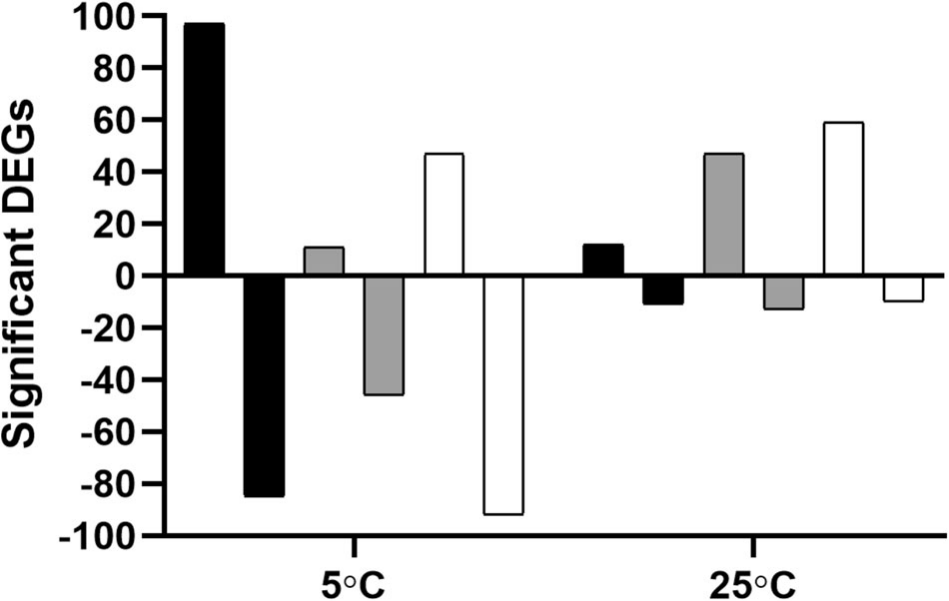
Gene regulation of *Campylobacter jejuni* post-exposure to chlorine or ASC. A single *C. jejuni* isolate was exposed to ASC or chlorine at either 5 or 25 °C and the RNA obtained was processed for RNA sequencing. The number of significantly regulated bacterial genes following exposure to chlorine (black) or ASC (gray) was computed by using *C. jejuni* exposed to 0.9% saline as a reference control. The regulated genes were also assessed in ASC treatment group (white) using chlorine as a control.

Chlorine is a commonly used sanitizer in the chicken meat industry but unlike ASC, its efficacy is linked with bacterial load and the presence of organic materials^16^. Here, the transcriptional effects of both the ASC and chlorine on *C. jejuni* were investigated at both 5 and 25 °C. Overall gene regulation was highly dependent on the treatment group (Fig. 2). A significantly higher (*P* ≤ 0.01) number of regulated genes were observed at 5 °C compared with 25 °C.

**Fig. 3:**
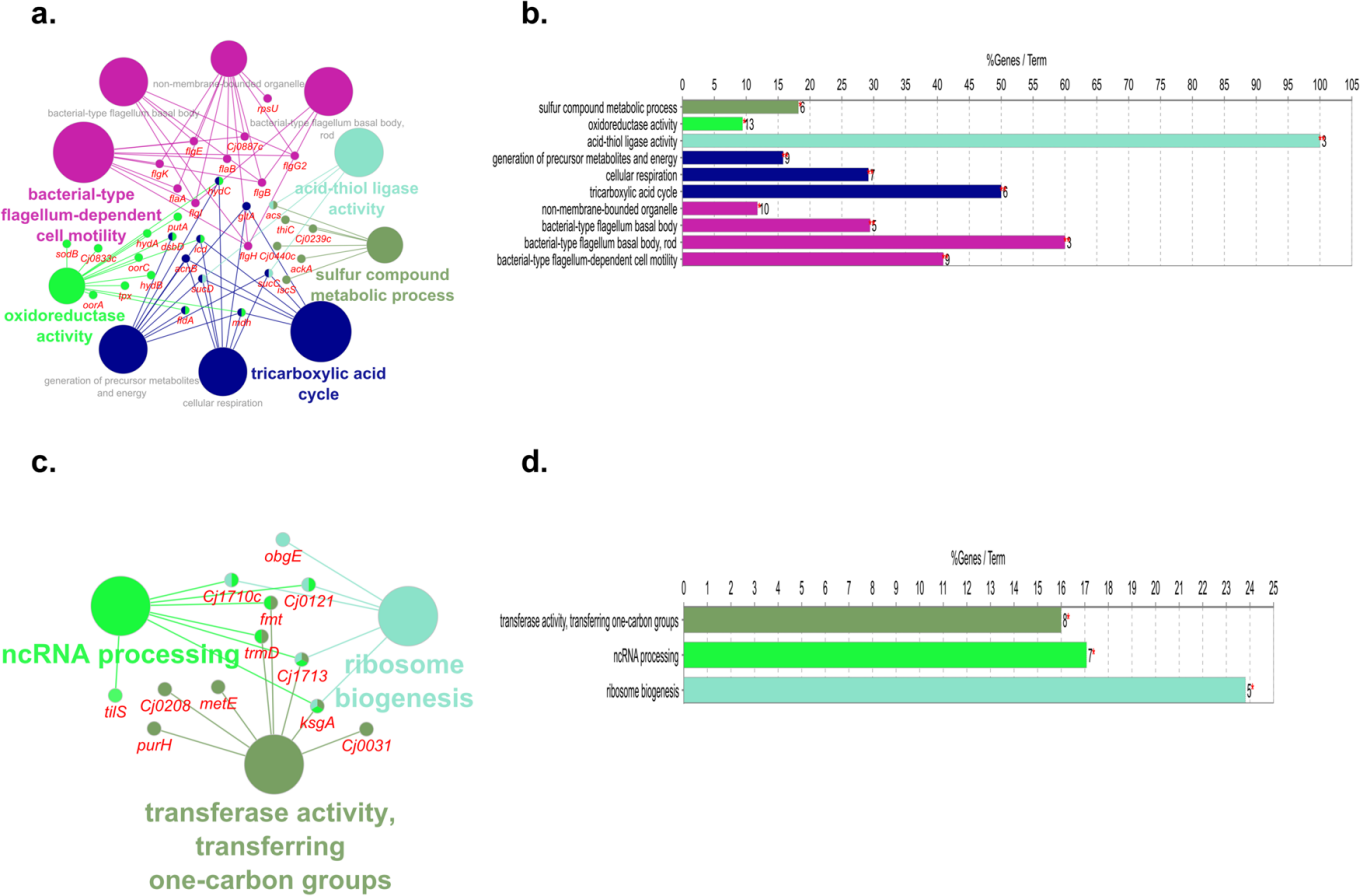
Biological pathway analysis of DEGs in exposure to chlorine at 5 °C. Pathways enriched by mapping the upregulated (**a**, **b**) DEGs obtained from *C. jejuni* post-exposure to chlorine at 5 °C. Pathways enriched by the downregulated (**c**, **d**) DEGs obtained from *C. jejuni* post-exposure to chlorine at 5 °C. Only significantly (*P* < 0.05) enriched pathways were obtained for biological pathway analysis of the regulated DEGs (FDR < 0.05; log_2_ fold change >1 or <−1) in ClueGO and CluePedia plugins in Cytoscape. In the graph, the length of individual bars indicates the percent of DEGs associated with respective terms out of the total associated genes.

Chlorine exposure at 5 °C resulted in the upregulation of 97 differentially expressed genes (DEGs), while 85 were downregulated. Biological pathway analysis of these genes showed that bacterial-type flagellum-dependent cell motility, tricarboxylic acid cycle (TCA cycle), cellular respiration, sulfur compound metabolic process, and acid-thiol ligase activity were activated (Fig. 3a, b). Pathway analysis of the downregulated genes showed that ribosome biogenesis, transferring activity, and ncRNA processing were inhibited by chlorine exposure at 5 °C (Fig. 3c, d). At 25 °C, 12 of the most significantly upregulated genes are involved in oxidative stress and ATP synthesis (Table S1). Genes related to flagellum assembly, catalase activity, and DNA metabolic process were downregulated (Table S1).

**Fig. 4:**
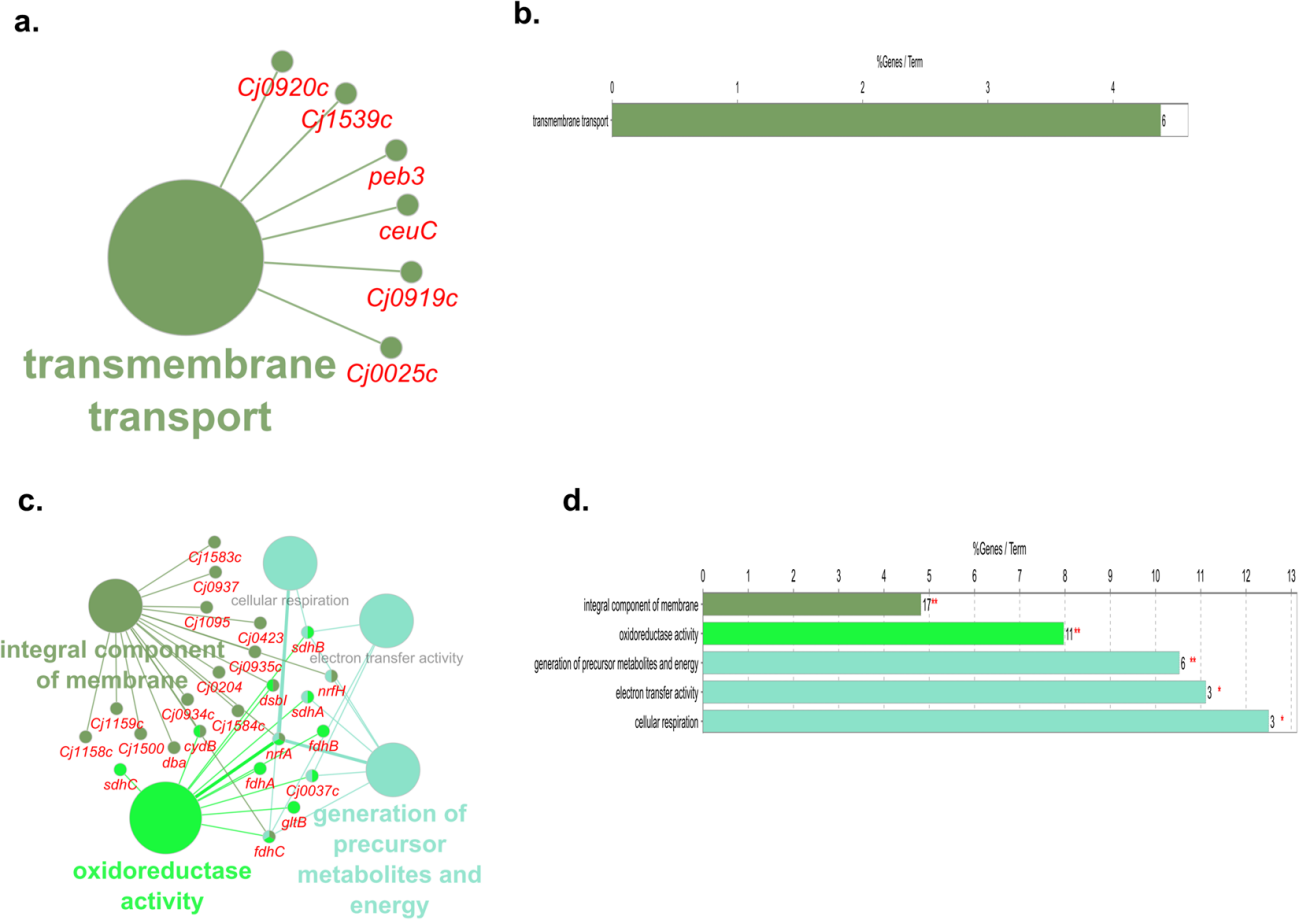
Biological pathway analysis of DEGs in exposure to ASC at 5 °C. Pathways enriched by mapping the upregulated (**a**, **b**) and downregulated (**c**, **d**) DEGs obtained from *C. jejuni* post-exposure to ASC at 5 °C. DEGs significantly (FDR < 0.05; log_2_ fold change >1 or <−1) regulated in *C. jejuni* in response to ASC treatment were mapped in ClueGO and CluePedia plugins in Cytoscape and only the significantly (*P* < 0.05) enriched biological pathways were visualized. In the graph, the length of individual bars indicates the percent of DEGs associated with respective terms out of the total associated genes.

Following exposure to ASC at 5 °C, 13 *C. jejuni* genes involved in transmembrane transport biological pathway were upregulated (Fig. 4a, b). Downregulated DEGs annotated to biological pathways including the generation of precursor metabolites and energy, oxidoreductase activity, and integral component of membrane (Fig. 4c, d). Exposure to ASC at 25 °C, resulted in the significant upregulation of 47 and downregulation of 13 DEGs that could not be analyzed for biological pathway analysis in ClueGO and CluePedia. Investigation of the individual upregulated DEGs revealed that most of these genes are involved in ion binding activity (Table S2). Increased expression of electron transport genes, catalytic and iron-sulfur cluster binding genes, as well as efflux protein genes was also observed. *C. jejuni* responded to oxidative stress through the upregulation of Cj0198c, which induces DNA replication and DNA repair through the involvement of *recR*. The downregulated DEGs included genes involved in flagellar motility and biosynthesis, periplasmic, and integral membrane protein activity (Table S3).

**Fig. 5:**
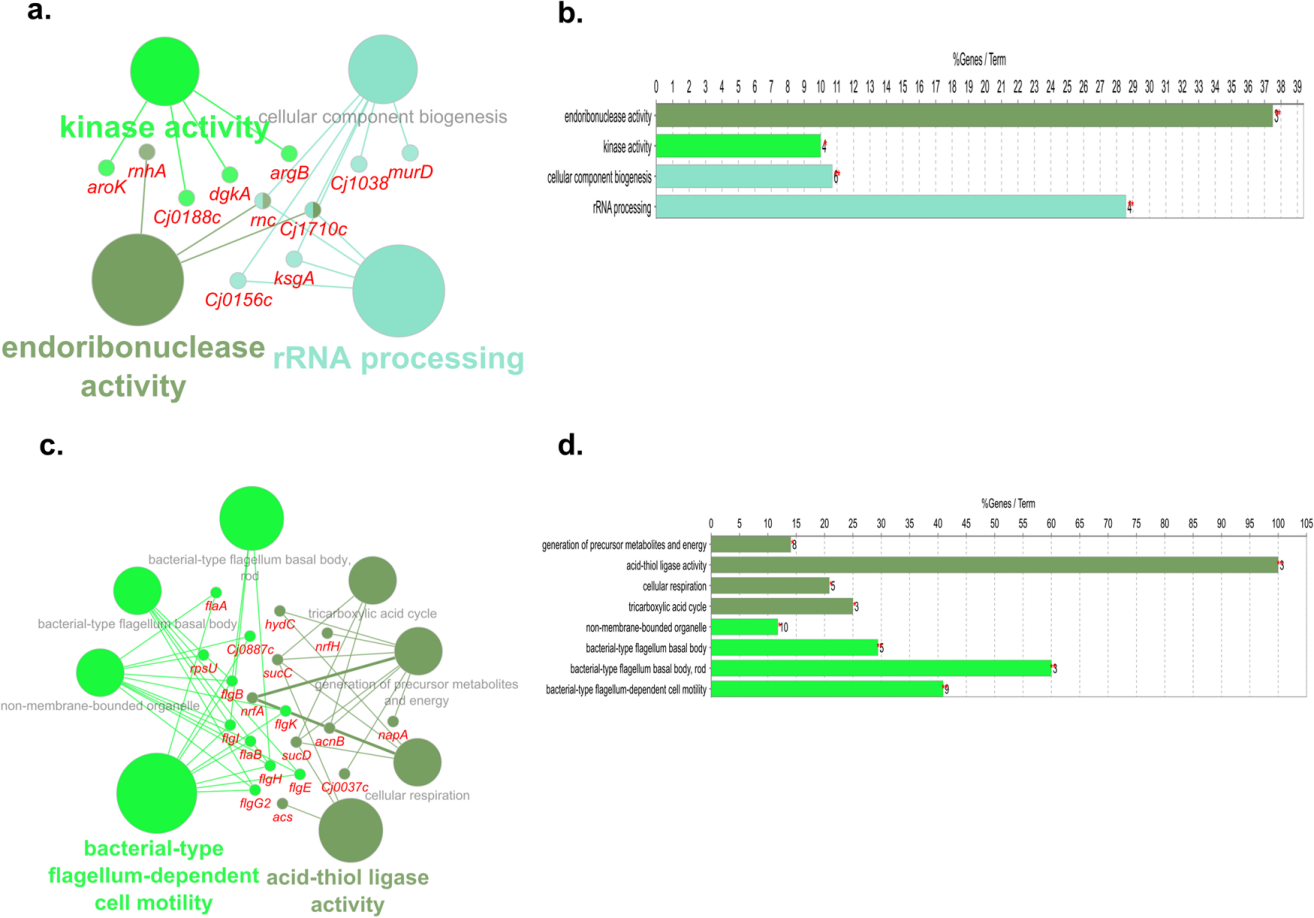
Biological pathway analysis of DEGs in exposure to ASC vs. chlorine at 5 °C. Pathways enriched by mapping the upregulated (**a**, **b**) and downregulated (**c**, **d**) DEGs at 5 °C. DEGs significantly (FDR < 0.05; log_2_ fold change >1 or <−1) regulated in *C. jejuni* were mapped in ClueGO and CluePedia plugins in Cytoscape and only significantly (*P* < 0.05) enriched biological pathways were visualized. In the graph, the length of individual bars indicates the percent of DEGs associated with respective terms out of the total associated genes.

**Fig. 6:**
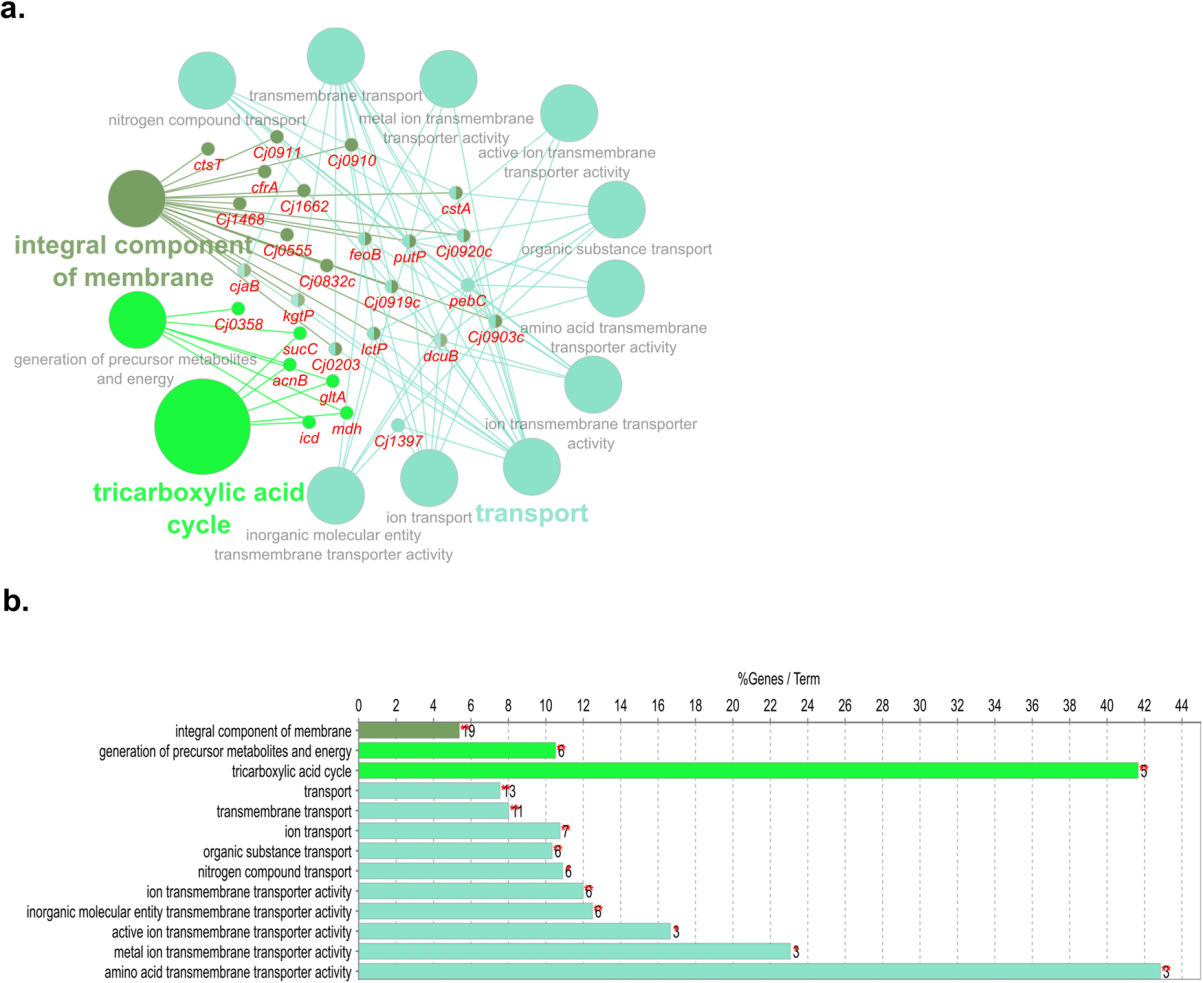
Biological pathway analysis of DEGs in exposure to ASC vs. chlorine at 25 °C. Pathways enriched by mapping upregulated (**a**, **b**) DEGs obtained from *C. jejuni* post-exposure to ASC at 25 °C. DEGs significantly (FDR < 0.05; log_2_ fold change >1 or <−1) upregulated in *C. jejuni* were mapped in ClueGO and CluePedia plugins in Cytoscape and only significantly (*P* < 0.05) enriched biological pathways were visualized. In the graph, the length of individual bars indicates the percent of DEGs associated with respective terms out of the total associated genes.

The transcriptomic response of *C. jejuni* exposed to ASC vs. chlorine was compared. At 5 °C, the majority of upregulated genes were found to be involved in rRNA processing, kinase activity, and endoribonuclease activity (Fig. 5a, b). Downregulated DEGs were annotated to biological pathway terms, such as bacterial flagella dependant cell motility and acid thiol ligase activity (Fig. 5c, d). At 25 °C, exposure to ASC resulted in 59 upregulated DEGs that annotated to pathway terms, including transport, TCA cycle, and integral membrane components (Fig. 6a, b). Downregulated DEGs (*n* = 10) play a role in flagellar motility and biosynthesis (Table S4).

To rule out temperature as a confounding factor, transcriptome data obtained from bacteria suspended in 0.9% saline at 5 and 25 °C were compared; a nonsignificant effect of temperature was observed.

**Fig. 7:**
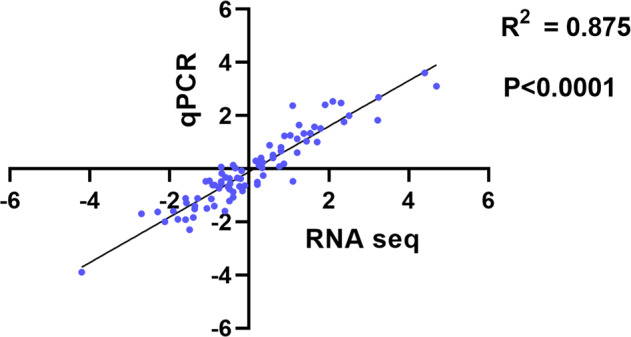
Linear regression analysis of RNA-Sequencing and qPCR data. The log_2_ fold change was calculated by comparing the control with treatment groups, the mean relative expression was calculated by 2^ΔΔcq^ method. Regression graph was generated in Graph Pad prism software.

Transcriptomic data were validated using qPCR. Primers used for RNAseq data validation showed high target specificity. The amplification efficiency of individual primers was in a range of 95–102% (Table S5). Regression analysis of the qPCR vs. the RNA-sequencing data showed a positive correlation (*P* < 0.0001; *R*^2^ = 0.878) (Fig. 7).

## Discussion

*Campylobacter* contamination of chicken meat remains a significant public health issue and its control is critical for the global poultry industry. ASC has previously been shown to be highly effective at reducing *Campylobacter* loads on chicken carcasses^17,22^ but little is known about its effects on bacterial physiology, cellular, and molecular responses. In this study, all *Campylobacter jejuni* isolates were inhibited at 7.03 ppm ASC demonstrating the effectiveness of this sanitizer at a far lower concentration than the recommended range of 500–1200 ppm^23^. These results were supported by time-kill and inactivation experiments, which showed that irrespective of exposure time and concentration, the culturability of the *C. jejuni* isolates was completely inhibited by low concentrations of ASC. Our results are in agreement with a previous investigation where foodborne pathogens exposed to 10 ppm of ASC were not resuscitated^24^. A similar kinetic has also been observed for the inactivation of *Salmonella* Typhimurium following exposure to 10 ppm ASC at pH 2.5^24^.

In this study, the high susceptibility of *C. jejuni* confirms the antimicrobial properties of ASC. Although the inactivation efficacy of chlorine is highly dependent on bacterial load^16^, our present findings with ASC revealed no effect of bacterial load. This could be due to the higher oxidizing capacity of the intermediate chemicals, ClO_2_ and chlorous acid, which are produced in ASC, compared with the hypochlorous acid produced in chlorine^25^. The presence of organic material (NB2 broth) also did not have an effect on bacterial culturability confirming the efficacy of ASC. To confirm these findings, field experiments are required, as the effectiveness of ASC in a commercial processing environment may vary due to the presence of organic matter and variable bacterial load.

The cellular response of *C. jejuni* after exposure to chlorine or ASC was further explored using transcriptomic analysis. Following exposure to either chlorine or ASC, more genes were significantly upregulated at 5 °C than 25 °C. The downregulation of genes was also higher at 5 °C than at 25 °C for all the treatment groups. This is likely due to the suppression of cellular activities in response to both the chemical and temperature stress.

Exposure of *C. jejuni* to chlorine induced several biological pathways including genes involved in bacterial-type flagellum-dependent cell motility and membrane proteins. Chlorine exposure has been shown to induce changes in membrane permeability and the morphology in *C. jejuni*^16^. Furthermore, oxidative phosphorylation in the cell membrane following chlorine exposure also leads to leakage of RNA, nucleotides, and proteins^26^ and increases energy requirements to compensate for the loss of intracellular components. Consistent with this, in the present study, *C. jejuni* genes involved in both the TCA cycle and cellular respiration pathways were upregulated after exposure to chlorine. *C. jejuni* also responded to oxidative stress by significantly upregulating genes involved in oxidoreductase activity including *sodB*, *oorAB*, *hydBC*, and *dsbD*. Pathway analysis of downregulated genes revealed that genes involved in ribosome biogenesis and ncRNA processing were downregulated by chlorine exposure at 5 °C suggesting that bacteria were exhibiting reduced cell growth, division, and replication caused by both temperature and chlorine induced stress. Similar responses have been shown in *Saccharomyces cerevisiae* after exposure to an alkylating agent, methyl methanesulfonate^27^. Although the DEGs were not expressed significantly at 25 °C compared to 5 °C, the cellular response of *C. jejuni* to chlorine caused by alteration of the membrane permeability was significant due to the upregulation of the genes encoding proteins involved in electron transport and iron-sulfur cluster binding (Cj1713, *fdxB*).

The response of *C. jejuni* to ASC was also assessed at 5 and 25 °C. Following exposure to ASC at 5 °C, C*. jejuni* upregulated DEGs involved in the activation of transmembrane transport biological pathways suggesting changes in bacterial membrane integrity and permeability^28,29^. Interestingly, the vital metabolic pathways in *C. jejuni* were suppressed. Genes encoding proteins involved in the generation of precursor metabolites and energy and cellular respiration (*fdhC* and *nrfA*) and oxidoreductase activity (*fdhABC*) were downregulated. This indicates a loss of cellular functions and survivability for *C. jejuni* after exposure to ASC.

The DEGs upregulated at 25 °C included proteins involved in reducing intracellular iron and the prevention of secondary oxidative DNA damage. In *Escherichia coli*, increased iron is important for the recovery of cells from stress^30^. Although *Campylobacter* lacks an SOS response in programmed cellular death^31^, in the current study, the upregulation of genes involved in DNA damage and repair (*recR* and Cj0198c) and efflux protein (Cj1174) indicated the cellular response mechanism in bacterial cell death. Activation of genes involved in DNA damage and repair has been noted in other bacteria treated with SH and hydrogen peroxide and PAA^28,32^.

To understand the effects of ASC at 5 and 25 °C, the data were analyzed using the chlorine exposure treatment as a control. The downregulation of DEGs involved in cellular metabolism and flagella-dependent cell motility at 5 °C indicated that oxidative injury was greater following the exposure to ASC. The upregulation of genes encoding proteins involved in rRNA processing and endoribonuclease activity indicated the ROS damaged DNA and RNA by ASC. Interestingly, genes encoding proteins involved in ROS induced bacterial survivability against the oxidative stress, *sodB* were not upregulated in the conditions applied in the current study. Downregulation of *katA* indicates that *C. jejuni* was not able to survive under oxidative stress induced by exposure to ASC at 5 °C. A similar response has been shown in SOS-induced cellular death in *Escherichia coli* following exposure to UV radiation^33^. Data from the 25 °C treatment group showed that *C. jejuni* was responding to the oxidative stress and the cellular damage was not as significant as recorded at 5 °C. The upregulation of genes involved in TCA cycle and membrane functions (*putP*, *dcuB*, *lctB*, and *febB*) indicated the oxidative response caused by membrane integrity changes. *C. jejuni* exhibited a similar stress response following exposure to ajoene^34^. Downregulated genes were mainly involved in flagellar motility and biosynthesis indicated the suppressed flagella activity by downregulation of cell membrane and chemotaxis activity. Our results indicated that oxidative cellular damage to *C. jejuni* was greater after exposure to ASC compared to chlorine and this was further enhanced at 5 °C.

In conclusion, we have demonstrated the sensitivity of *Campylobacter jejuni* to ASC. In ASC, *C. jejuni* experienced severe, irreversible injury that ultimately resulted in bacterial cell death and lysis. ASC induced lethal oxidative injury in *C. jejuni* by inhibiting all vital cellular functions that were further aggravated at low temperatures. In contrast, chlorine induced an adaptive stress response mechanism enabling *C. jejuni* to survive oxidative stress. The transcriptomic data presented here represent gene expression of the surviving cell population, which could include a range of states of bacterial cell death and survival. Outcomes of these experiments have significant implications for food safety and public health. Further studies, however, are necessary to investigate the stress response mechanisms in *C. jejuni* enabling them to survive chemical stress in the food chain, and how this affects bacterial virulence. Additional investigation is also required to identify optimal points in poultry meat processing to utilize ASC for the control of *Campylobacter* spp.

## Methods

### *Campylobacter* isolates

*Campylobacter jejuni* (*n* = 89) isolates previously cultured from broiler carcasses^17^ were selected for this study. Species identification was confirmed previously^16^. *Campylobacter jejuni* ATCC 33291 was included as a control strain. Pure cultures were maintained long-term at −80 °C in a 50:50 mixture of brain heart infusion broth and 100% glycerol. Isolates were resuscitated on sheep blood agar (SBA) (ThermoFisher Scientific, Australia) and incubated at 42 °C in 10% CO_2_ for 48 h.

### MIC and MBC of acidified sodium chlorite

The MIC of ASC was determined using the broth micro-dilution method in NB2 (ThermoFisher Scientific, Australia) according to the Clinical and Laboratory Standard Institute guidelines^35^. *C. jejuni* ATCC 33291 was included as an experimental control. NB2 was acidified to pH 2.5 using 4 M citric acid (Sigma Aldrich, USA). Dilutions of ASC (3600–7.03 ppm) were prepared using 31% sodium chlorite (ChemSupply, Australia) in acidified NB2. *Campylobacter* inoculums were prepared using a 0.5 McFarland standard and confirmed by measuring the optical density at 600 nm to obtain 10^8^ CFU/mL. To determine MIC, 10 μL of the bacterial culture was inoculated into 990 μL of ASC in 96-well round-bottom micro-titre plates. NB2 without ASC and containing *Campylobacter* was a positive control and NB2 only was the negative control. MIC plates were incubated at 42 °C in 10% CO_2_ for 20 h. The lowest concentration that did not give visible *Campylobacter* growth was defined as the MIC. Isolates were tested in duplicate and the assay was repeated twice.

Ten μL of broth from each well-showing growth inhibition was drop plated onto SBA plates and incubated at 42 °C in 10% CO_2_ for 20 h to determine the MBC. The MBC was defined as the lowest bactericidal concentration of ASC required to kill *Campylobacter*.

### Time-kill assay

Time-kill experiments were performed to characterize *C. jejuni* susceptibility to three concentrations (225, 450, and 900 ppm) of ASC in the presence of organic materials. Seven *C. jejuni* isolates were randomly selected for these experiments. NB2 or 0.9% saline was acidified with 4 M citric acid to pH 2.5 before the addition of ASC. The bacterial inoculum was prepared in 0.9% saline as described above. Overall, 10 μL of inoculum was added into 990 μL of each ASC dilution to obtain 10^6^ CFU/mL. Experiments were conducted at both 5 and 25 °C. Bacterial counts were determined at multiple time points post exposure (1, 5, 15, 30, 45, 60 min). Serial tenfold dilutions in 0.9% saline were prepared and 10 μL of each dilution was drop plated onto SBA and incubated at 42 °C in 10% CO_2_. Colonies were enumerated and reported as CFUs/mL. Each treatment group contained three biological replicates and the experiments were repeated twice.

### Inactivation of *C. jejuni* in ASC

Inactivation assays and time-kill experiments were conducted to further characterize the sensitivity of *C. jejuni* isolates to ASC. ASC concentrations ranging from 900 to 1.76 ppm were prepared in acidified 0.9% saline (pH 2.5). To determine the effect of *Campylobacter* load, two inoculum doses, 10^6^ and 10^8^ CFU/mL were exposed to ASC for 1 min at either 5 or 25 °C. The bacterial count was obtained by drop plating 10 μL of serial tenfold dilutions onto SBA plates and incubating at 42 °C in 10% CO_2_ for 48 h. Inactivation experiments were performed in triplicate and repeated twice. Following exposure, 100 μL of each treatment and control groups were inoculated into 900 μL Preston broth (NB2 with *Campylobacter* selective supplement) (ThermoFisher Scientific, Australia) and incubated at 42 °C in 10% CO_2_ for resuscitation. After 24 and 48 h of incubation, 10 μL from each treatment group was drop plated onto SBA and incubated at 42 °C in 10% CO_2_ for 48 h. The resuscitation experiment was performed in triplicate and repeated twice.

### *Campylobacter jejuni* RNA extraction for RNA sequencing and qPCR

To study the transcriptional regulation of *Campylobacter* in response to chlorine and ASC exposure, a single *C. jejuni* isolate was incubated in NB2 at 42 °C with 10% CO_2_ for 48 h with agitation at 80 rpm. The broth culture was centrifuged at 4300 × *g* for 20 min. Pellets were resuspended in 0.9% saline to obtain 10^9^ CFU/mL *Campylobacter*. Chlorine (8 ppm), ASC (900 ppm), and 0.9% saline solutions were prepared as described earlier. *C. jejuni* was exposed to chlorine and ASC for 2 and 1 min, respectively at 5 or 25 °C. These times reflect exposure times that are used in the poultry meat industry. In the control group, *C. jejuni* was exposed to 0.9% saline for 2 min. Three biological replicates were included in each treatment. After exposure, samples were centrifuged at 4300 × *g* for 8 min to pellet *C. jejuni*.

Total RNA was extracted using Trizol (Invitrogen, USA). Bacterial pellets were resuspended in lysis buffer composed of 30 μL lysozyme (100 mg/mL) (Merk, Australia), 50 μL proteinase K (20 mg/mL) (Qiagen, Australia), 2.5 μL *β*-mercaptoethanol (Merk, Australia), 5 μL of 10% SDS (Invitrogen, USA), and 112.5 μL of 1 M Tris EDTA buffer (Merk, Australia). Samples were incubated for 15 min at 37 °C and 20 μL of 3 M sodium acetate (Thermo Scientific, Australia) was added followed by 750 μL Trizol. The samples were mixed and incubated on ice for 5 min. To separate the RNA containing phase from phenol, 250 μL chloroform (Merk, Australia) was added. The samples were mixed, and incubated on ice for 3 min and then centrifuged at 20,000 × *g* for 15 min at 4 °C. The top 300–350 μL of the aqueous layer was transferred into RNAse free centrifuge tubes. To precipitate RNA, 500 μL of chilled 2-propanol was added and the samples were incubated at −20 °C for 1 h. RNA pellets were obtained by centrifugation at 20,000 × *g* for 15 min at 4 °C. Pellets were washed twice with 75% ethanol and allowed to dry on ice for 20 min before resuspension in 20 μL RNAse free water and were then processed for DNAse digestion (RNase Free DNase Set; Qiagen, Australia) and RNA cleanup (Monarch RNA Cleanup Kit; New England Biolab, USA) as per the manufacturers’ protocols. RNA quality and integrity were assessed using an Agilent RNA ScreenTape System in TapeStation 2200 (Agilent Technologies, Germany). RNA sequencing was performed by the Australian Genome Research Facility, Melbourne, Victoria. RNA sequencing and analysis were performed as described previously^36^ with modifications.

### cDNA synthesis and library preparation

For cDNA library preparation, mRNA was fragmented into small pieces using heat and divalent cations with the Illumina TruSeq Stranded mRNA Library prep kit. First-strand cDNA was prepared using Super Script II Reverse transcriptase (Invitrogen, Australia) and a master mix containing dATP, dGTP, dCTP, and dUTP was used in the second strand cDNA preparation as per the manufacturer’s protocol.

Libraries were prepared by 3′-adenylation of cDNA fragments and amplification of the library by PCR. Libraries were pooled and clustered through the Illumina cBot system using TruSeq PE Cluster Kit v3 reagents followed by sequencing of 100 bp single read on the Illumina NovaSeq 6000 system with TruSeq SBS Kit v3 reagents. The primary sequence data were generated using the Illumina bcl2fastq 2.20.0.422 pipeline using the standard FASTQ format.

### Reads mapping and transcriptome assembly

Cleaned sequence reads were aligned against the reference genome of *Campylobacter jejuni* ATCC 700819 (National Center for Biotechnology Information (NCBI) RefSeq GCF_000009085.1) using the STAR aligner v2.5.3a^37^. The feature summary table was created providing an overall mapping rate with the genome end and genome features (feature = exons). The counts of reads mapping to each known gene were summarized at the gene level using featureCounts v1.5.3^38^. Digital gene expression values or raw gene counts were used in edgeR V3.26.5^39^ of R packages v3.6.0 for computing differential gene expression in counts per million (CPM). Transcripts were assembled with the StringTie tool v1.3.3^40^. Reads alignments and reference annotation-based transcript assembly (RABT) option generated assembly for known and potentially novel transcripts^41^.

### Differential gene expression analysis

CPM values were used in edgeR to compute the DEGs in *C. jejuni* exposed to sanitizers at 5 and 25 °C. During the analysis, the default trimmed mean of M values normalization method of edgeR was used to normalize the counts between the treatment groups. For assessing the regulation of genes of *C. jejuni* affected by chlorine and ASC, the control group was used a reference at 5 and 25 °C, General Linear Model was used to quantify the DEGs.

### Functional annotation of significantly regulated DEGs

The DEGs with log_2_ fold change ≥1 or ≤−1 and false discovery rate ≤0.05 were considered to be significantly regulated and were analyzed for functional annotation in ClueGO v2.5.6^42^. and CluPedia^43^ plugins in Cytoscape v3.7.2. The DEGs were enriched for terms specific to *Campylobacter jejuni*: biological process, cellular component, and molecular function pathways. In ClueGO, significantly enriched biological pathways were identified by the two-sided hypergeometric test with *P* < 0.05 based on Benjamini–Hochberg.

### RNA-Sequencing data validation by qPCR

Primers for candidate target and reference genes were designed using the NCBI software (Table S5). Ten genes that were significantly up or downregulated following the exposure to chlorine or ASC were selected. *16S rRNA*, *glyA*, and *bioD* were selected as reference genes due to their stable expression under different treatment conditions. Primers were optimized for target specificity using qPCR, melt curve analysis, and products were visualized by gel electrophoresis with 2% agarose gel. For determining the amplification efficiency of individual primers, qPCR was performed on eight different fivefold serial dilutions of *C. jejuni* cDNA.

From individual RNA samples (*n* = 18), ~100 ng was reversely transcribed to cDNA using the QuantiTect Reverse Transcription Kit (Qiagen, Australia) as per the manufacturer’s protocol. For qPCR, using SensiFast Sybr Hi-Rox Kit (Bioline, Australia), mastermix was prepared and loaded into 384 well plates by epMotion 5075 Eppendorf robot (Eppendorf, USA). Quantitative PCR was conducted in duplicate using a 20 µL reaction volume. Thermocycling conditions in QuantStudio 6 (ABI, Australia) thermal cycler were: polymerase activation at 95 °C for 3 min, 40 cycles of denaturation at 95 °C for 15 s, annealing either at 58 or 60 °C for 30 s and extension at 72 °C for 30 s. A melting curve step was included to assess the specificity of amplification. For relative expression data analysis, the expression levels of the candidate target genes were normalized against the average of the reference genes *16S rRNA*, *glyA*, and *bioD*.

### Statistical analysis

*Campylobacter* count data were analyzed in GraphPad Prism Version 8.3.0 (GraphPad Software, Inc., USA) using one- and two-way analysis of variance with Tukey’s multiple comparisons test. *P* values <0.05 were considered statistically significant. The relative expression data of individual genes were analyzed by 2^−ΔΔCq^ method from quantitation cycle (C_q_) values using the reference genes *16S rRNA*, *glyA*, and *bioD*. qPCR data were expressed as log_2_ fold change.

### Reporting summary

Further information on research design is available in the Nature Research Reporting Summary linked to this article.

## Supplementary information

Reporting Summary

Supplementary Information

## Data Availability

The RNA sequence raw data (fastq files) have been deposited at the National Center for Biotechnology Information (NCBI), Sequence Read Archive (SRA) under the BioProject accession number PRJNA672226.
