# Supplementary material for: Transcriptomic response of Campylobacter jejuni following exposure to acidified sodium chlorite
Source: NPJ Sci Food. 2021 Aug 2;5:23. doi: 10.1038/s41538-021-00103-5 (PMC8329286; doi:10.1038/s41538-021-00103-5)
Supplement: Supplementary file 2 — Supplementary Information [file 41538_2021_103_MOESM2_ESM.pdf]

Table S1. Significant DEGs of *Campylobacter jejuni* in response to chlorine treatment at 25°C

Gene Symbol	Gene Name	Fold Change	FDR	Known function
rpsl	30S ribosomal protein S9	+4.00	1.429E-05	Structural constituent of ribosome, ribosome, translation
**Cj0353c**	Phosphatase	+1.39	1.041E-06	Exopolyphosphatase activity,
**Cj1713**	Hypothetical protein Cj1713	+1.31	0.017	catalytic activity, iron-sulfur cluster binding
**Cj0295**	Putative acetyltransferase	+1.24	0.000	N-acetyltransferase activity, metabolic process
fdxB	Putative ferredoxin	+1.21	1.429E-05	Electron transport, iron-sulfur cluster binding
**Cj1095**	Putative integral membrane protein	+1.18	0.003	nitrogen compound metabolic process
atpH	ATP synthase F1 sector delta subunit	+1.05	0.001	Hydrogen-transporting two-sector ATPase activity
**Cj0140**	Hypothetical protein Cj0140	+1.04	0.000	McrBC 5-methylcytosine restriction system component
**Cj0126c**	Hypothetical protein Cj0126c	+1.01	0.0205	Uncharacterized conserved protein
hemH	Putative ferrochelatase	+1.04	0.005	Ferrochelatase activity, heme biosynthetic process,
**Cj0176c**	Putative lipoprotein	+1.04	0.001	Unknown function
**Cj0566**	Hypothetical protein Cj0566	+1.77	0.026	Unknown function
**Cj0040**	Hypothetical protein	-4.35	0.023	Sugar: hydrogen symporter activity
**Cj1465**	Hypothetical protein	-1.74	2.248	Flagellum assembly, flagellum
pebC	ABC-type amino-acid transporter ATP-binding protein	-1.25	0.000	ATP binding, DNA metabolic process, DNA repair, ATPase activity
flgM	Hypothetical protein	-1.25	0.000	Transcription repressor activity, flagellum
hrcA	putative heat shock regulator	-1.23	0.000	Transcriptional regulator of heat shock gene
**Cj1631c**	Hypothetical protein	-1.17	0.001	Calcium ion binding
**Cj0919c**	Putative ABC-type amino-acid transporter permease protein	-1.17	0.004	Transporter activity, Gram-negative-bacterium-type cell wall
kgtP	Alpha-ketoglutarate permease	-1.02	0.002	Transporter activity, integral to membrane
katA	Catalase	-1.01	0.000	Catalase activity, iron ion binding, electron transport
ctsT	Putative periplasmic protein	-4.79	0.024	Unknown function
**Cj1386**	Ankyrin-repeat containing protein	-1.16	0.006	Unknown function

The expressed genes with $\log_2$ fold change and their specific functions is shown. Positive (+) and negative (-) signs show up-regulated and down-regulated genes, respectively. The functions of the enlisted genes were sourced from the genecard data base. The FDR (false discovery rate) is mentioned to show the significance of the DEGs

Table S2. Up-regulated DEGs of *Campylobacter jejuni* in response to ASC treatment at 25°C

Gene Symbol	Gene Name	Fold change	FDR	Possible function
tRNALeu	TRNA-Leu	+2.04	0.001	Aminoacyl-tRNA biosynthesis
**Cj0241c**	Putative iron-binding protein	+1.93	0.025	Metal ion binding
**Cj0911**	Putative periplasmic protein	+1.86	0.000	Electron transport, antioxidant activity, oxidoreductase activity
**Cjr07**	16S ribosomal RNA	+1.78	0.000	Ribosome
**Cj0021c**	Hypothetical protein	+1.57	0.000	Catalytic activity
**Cj1582c**	Putative peptide ABC-transport system permease protein	+1.52	0.036	Transporter activity
tRNAGln	TRNAGln	+1.47	0.005	Aminoacyl-tRNA biosynthesis
**Cj0967**	Putative periplasmic protein	+1.46	0.013	Zinc ion binding
**Cj0198c**	Helicase-like protein	+1.44	0.046	DNA binding, ATP binding, DNA replication, DNA repair
**Cj0916c**	Hypothetical protein Cj0916c	+1.42	0.002	Uncharacterized small protein
**Cjr08**	23S ribosomal RNA	+1.42	8.6E-06	Ribosome
**Cj0555**	Putative integral membrane protein	+1.40	2.4E-06	Di- and tricarboxylate transporters
cstA	Carbon starvation protein A homolog	+1.38	6.1E-07	Cellular response to starvation
**Cj1095**	Putative integral membrane protein	+1.36	0.001	Nitrogen compound metabolic process
**Cj0909**	Putative periplasmic protein	+1.33	3.7E-05	Uncharacterized protein conserved in bacteria
**Cj1397**	Hypothetical protein Cj1397	+1.30	0.021	Iron ion binding
**Cj0919c**	Putative ABC-type amino-acid transporter permease protein	+1.29	0.000	Transporter activity, Gram-negative-bacterium-type cell wall
**Cj1113**	Hypothetical protein Cj1113	+1.27	0.021	Oxidoreductase activity, transition metal ion binding
cysQ	CysQ protein homolog	+1.27	0.001	Inositol or phosphatidylinositol phosphatase activity
**Cj0920c**	Putative ABC-type amino-acid transporter permease protein	+1.25	3.7E-05	Transporter activity, Gram-negative-bacterium-type cell wall
**Cj1174**	Putative efflux protein	+1.24	0.014	Membrane transporters of cations and cationic drugs
recR	Recombination protein	+1.22	0.045	DNA repair
**Cj1608**	Possible two-component regulator	+1.21	0.009	Two-component response regulator activity
**Cj0358**	Putative cytochrome C551 peroxidase	+1.21	0.000	Iron ion binding, heme binding electron transport
**Cj0125c**	DksA-like protein	+1.18	0.003	zinc ion binding
rpmB	50S ribosomal protein L28	+1.18	6.9E-05	Structural constituent of ribosome
dnaJ	Chaperone DnaJ	+1.17	0.003	ATP binding, protein folding
cjaB	CjaB protein	+1.16	0.000	Integral component of membrane

**Cj0984**	Hypothetical protein Cj0984	+1.15	0.000	Uncharacterized protein conserved in bacteria
**Cj1662**	Putative integral membrane protein	+1.15	0.003	Membrane
**Cj0833c**	Oxidoreductase	+1.15	0.000	Metabolic process, oxidoreductase activity
kgtP	Alpha-ketoglutarate permease	+1.14	0.000	Transporter activity
**Cj0126c**	Hypothetical protein Cj0126c	+1.12	0.010	Uncharacterized conserved protein
gltA	Citrate synthase	+1.10	1.2E-05	Citrate (Si)-synthase activity
modC	Putative molybdenum transport ATP-binding protein	+1.10	0.01	ATP binding, sulfate transmembrane-transporting ATPase activity
tRNASer	tRNASer	+1.09	0.000	Aminoacyl-tRNA biosynthesis
**Cj1169c**	Putative periplasmic protein	+1.09	0.001	Unknown function
**Cj0363c**	Putative oxidoreductase	+1.06	0.000	Catalytic activity, iron-sulfur cluster binding
**Cj0140**	Hypothetical protein Cj0140	+1.06	0.001	McrBC 5-methylcytosine restriction system component
rpsI	30S ribosomal protein S9	+1.05	9.3E-06	Structural constituent of ribosome
trmA	tRNA (uracil-5-)-methyltransferase	+1.03	0.012	tRNA processing
**Cj0849c**	Hypothetical protein Cj0849c	+1.01	0.008	Flagellum assembly, bacterial-type flagellum hook
rplM	50S ribosomal protein L13	+1.00	0.000	Structural constituent of ribosome
**Cj0834c**	Ankyrin repeat-containing possible periplasmic protein	+1.00	1.6E-06	Cellular amino acid metabolic process
**Cj0176c**	Putative lipoprotein	+3.43	0.007	Unknown function
**Cj0910**	Putative periplasmic protein	+1.41	0.000	Unknown function
**Cj0692c**	Putative periplasmic protein	+1.18	0.018	Unknown function

The expressed genes with log_2 fold change and their specific functions is shown. The functions of the enlisted genes were sourced from the genecard data base. The FDR is mentioned to show the significance of the DEGs. Positive sign (+) shows up-regulated genes.

Table S3. Down-regulated DEGs of *Campylobacter jejuni* in response to ASC treatment at 25°C.

Gene Symbol	Gene Name	Fold change	FDR	Possible function
**Cj1200**	Putative periplasmic protein	-2.13	0.011	ABC-type metal ion transport system, periplasmic component
metE	5-methyltetrahydropteroyltriglutamate--homocysteine methyl transferase	-1.97	9.658E-05	5-methyltetrahydropteroyltriglutamate-homocysteine S-methyltransferase activity and zinc ion binding
**Cj1199**	Putative iron/ascorbate-dependent oxidoreductase	-1.62	0.002	Iron ion binding, oxidoreductase activity
**Cj1533c**	Putative helix-turn-helix containsing protein	-1.22	0.000	Predicted ATPase (AAA+ superfamily)
**Cj0729**	Hypothetical protein Cj0729	-1.21	0.008	Hydrolase activity, nucleotide metabolic process
flhB	Flagellar biosynthesis protein FlhB	-1.07	1.545E-05	Flagellar biosynthesis
flgG2	Flagellar basal-body rod protein	-1.05	5.515E-06	Ciliary or flagellar motility, motor activity
leuA	2-isopropylmalate synthase	-1.05	0.002	2-isopropylmalate synthase activity, leucine biosynthetic process
**Cj0830**	Putative integral membrane protein	-1.03	2.659E-05	Uncharacterized protein conserved in bacteria
cbpA	Putative curved-DNA binding protein	-1.01	0.000	Heat shock protein binding, unfolded protein binding
**Cj0200c**	Putative periplasmic protein	-1.31	0.014	Unknown function
**Cj1356c**	Putative integral membrane protein	-1.13	0.001	Unknown function
**Cj0748**	Hypothetical protein Cj0748	-1.08	0.020	Unknown function

The expressed genes with log_2 fold change and their specific functions is shown. The functions of the enlisted genes were sourced from the genecard data base .The FDR is mentioned to show the significance of the DEGs. Negative sign (-) shows up-regulated genes.

Table S4. Down-regulated DEGs of *Campylobacter jejuni* in response to ASC treatment versus chlorine (control) at 25°C

Gene Symbol	Gene Name	Fold Change	FDR	Possible Function
**Cj1200**	Putative periplasmic protein	-1.90	0.027	ABC-type metal ion transport system, periplasmic component
metE	5-methyltetrahydropteroyltriglutamate-Homocysteine methyltransferase	-1.76	0.000	5-methyltetrahydropteroyltriglutamate-homocysteine S-methyltransferase activity
**Cj0889c**	Putative sensory transduction histidine kinase	-1.11	0.002	Signal transduction histidine kinase,two-component sensor activity, ATP binding
**Cj1276c**	Putative integral membrane protein	-1.08	6.342E-05	Cell division protein, membrane
flgG2	Flagellar basal-body rod protein	-1.05	1.756E-06	Ciliary or flagellar motility, motor activity, structural molecule activity, bacterial-type flagellum
flhB	Flagellar biosynthesis protein FlhB	-1.01	1.352E-05	Flagellar biosynthesis pathway, component FlhB
**Cj1162c**	Hypothetical protein	-1.38	0.022	Unknown function
**Cj0748**	Hypothetical protein	-1.25	0.006	Unknown function
**Cjr09**	5S ribosomal RNA	-1.13	1.107E-06	Unknown function
**Cj0651**	Putative integral membrane protein	-1.77	0.000	Unknown function

The expressed genes with log_2 fold change and their specific functions is shown. The functions of the enlisted genes were sourced from the genecard data base .The FDR is mentioned to show the significance of the DEGs. Negative sign (-) shows up-regulated genes.

Table S5. Primer sequence used in qPCR for RNA-Sequencing data validation

Gene symbol	Primer sequence (5'-3')	Fragment size (bp)	NCBI Accession number	Annealing (°C)	qPCR efficiency (%)	Correlation coefficient (R^2)	Slope
*16S rRNA**	**F**:TGCTAGAAGTGGATTAGTGG<br>**R**: GTATTAGCAGTCGTTTCCAA	91	NR_041834.1	58	98	0.992	-3.37
*glyA**	**F**:ACGGCGTAGAACTTGATGGA<br>**R**:TCACCTGCCACAACAAGACC	202	NC_002163.1	58	96	0.99	-3.42
*bioD**	**F**:TCAAGCAGGAACTCCAACAGA<br>**R**:TCCAAGATGAGGAGAAGCTGG	109	NC_002163.1	58	100	0.991	-3.29
cstA	**F**:TGGTGGTTGCTGCTGTATGT<br>**R**:GTGCTGGTGTTGCACGATTT	105	NC_002163.1	58	101	0.991	-3.30
thiC	**F**:CTTTGCGTCCTGGCTCTACT<br>**R**:AATCCCAACATCCCAAGCCC	101	NC_002163.1	58	96	0.996	-3.41
sucD	**F**:GGTTGCGTGGGGCTTATTTC<br>**R**:TAGCCGCTTCCACTTCCAAA	223	NC_002163.1	58	98	0.996	-3.36
Cj1200	**F**:TTTACCGGTTTTGCATGGGC<br>**R**:ACAAGATGGGTGCCTTGTCT	242	NC_002163.1	58	96	0.985	-3.43
fdhC	**F**:CGCAGCGATTTCTTGGGTG<br>**R**:CTGCAGGTACAGGACGTTTGA	248	NC_002163.1	58	96	0.995	-3.42
ksgA	**F**:CCATCAAGACGCAAGCGAAG<br>**R**:CAGCCATTTCCCTTTGTGCC	161	NC_002163.1	58	95	0.997	-3.44
fdxB	**F**:ACTGCGTATGTTGTGATGCTTG<br>**R**:GCAACAATACAGGCAGGCTC	136	NC_002163.1	60	102	0.998	-3.27
flgH	**F**:GCACCAGGATCGCTTTTTGG<br>**R**:CTGTTAAAGCACCTCCGCCT	175	NC_002163.1	60	99	0.998	-3.34
Cj0920c	**F**:AAGCCCTTTTGCGGTATGGA<br>**R**:CTTGTAGCCATAACTCCGCCT	147	NC_002163.1	60	97	0.995	-3.38

Candidate reference (*) and target genes in expression studies by qPCR. To calculate the amplification efficiency of individual primers, a standard curve was generated using a 5-fold dilution of cDNA amplified in Quant Studio 6 (ABI, Australia) thermocycler real-time system. Standard curve was obtained by plotting the Cq values against the log of the starting quantity of template for each dilution.
